# Iron elevation and adipose tissue remodeling in the epididymal depot of a mouse model of polygenic obesity

**DOI:** 10.1371/journal.pone.0179889

**Published:** 2017-06-26

**Authors:** Xiaoya Ma, Vinh T. Pham, Hiroyuki Mori, Ormond A. MacDougald, Yatrik M. Shah, Peter F. Bodary

**Affiliations:** 1 School of Kinesiology, University of Michigan, 1402 Washington Hts., Ann Arbor, MI, United States of America; 2 Department of Molecular & Integrative Physiology, Ann Arbor, MI, United States of America; 3 Internal Medicine, University of Michigan Medical School, Ann Arbor MI, United States of America; University of Tampere, FINLAND

## Abstract

**Background:**

Iron dysregulation is a potential contributor to the pathology of obesity-related metabolic complications. KK/HIJ (KK) mice, a polygenic obese mouse model, have elevated serum iron levels. A subset of KK male mice display a bronzing of epididymal adipose tissue (eAT) associated with >100-fold (p<0.001) higher iron concentration.

**Methods:**

To further phenotype and characterize the adipose tissue iron overload, 27 male KK mice were evaluated. 14 had bronzing eAT and 13 had normal appearing eAT. Fasting serum and tissues were collected for iron content, qPCR, histology and western blot.

**Results:**

High iron levels were confirmed in bronzing eAT (High Iron group, HI) versus normal iron level (NI) in normal appearing eAT. Surprisingly, iron levels in subcutaneous and brown adipose depots were not different between the groups (p>0.05). The eAT histology revealed iron retention, macrophage clustering, tissue fibrosis, cell death as well as accumulation of HIF-2α in the high iron eAT. qPCR showed significantly decreased *Lep* (leptin) and *AdipoQ* (adiponectin), whereas *Tnfα* (tumor necrosis factor α), and *Slc40a1* (ferroportin) were up-regulated in HI (p<0.05). Elevated HIF-2α, oxidative stress and local insulin signaling loss was also observed.

**Significance:**

Our data suggest that deposition of iron in adipose tissue is limited to the epididymal depot in male KK mice. A robust adipose tissue remodeling is concomitant with the high iron concentration, which causes local adipose tissue insulin resistance.

## Introduction

Iron dysregulation is a potential contributor to the pathology of obesity-related metabolic complications, such as type 2 diabetes mellitus (T2DM). Human studies have demonstrated elevated iron stores to precede insulin resistance [1, 2], while lowering serum iron can increase insulin sensitivity [3]. This association between iron and diabetes is hypothesized to induce inflammation and promote oxidative stress [4, 5]. In a polygenic obese and diabetic mouse model (KKA^y^ mice) iron chelation resulted in an amelioration of adipocyte hypertrophy by suppressing oxidative stress, inflammatory cytokines, and macrophage infiltration in the epididymal fat depot [6]. Adiponectin, an insulin-sensitizing adipokine secreted from adipocytes, is inversely associated with adipose tissue mass. Studies in humans, mice and cultured cells have demonstrated that excessive iron lowers adiponectin production and increases diabetes risk [7]. Both an increase in inflammatory cytokines and a decrease in adiponectin, can lead to the interruption of insulin signaling pathways and consequently to a decrease in insulin sensitivity [8–11]. In addition, strategies to reduce iron concentration (e.g. low iron diet, chelation therapy, and phlebotomy) have led to improvements in insulin sensitivity in obese animal models [6, 12, 13] and humans [3, 7, 14]. To this end, iron has been demonstrated to play an important role in metabolic syndrome, including detrimental effects on the modulation of metabolism through inflammation and oxidative stress. However, it should be noted that most of these studies are based on association and that the mechanisms linking increased iron stores to insulin resistance still need to be determined.

Adipocytes express common regulators of iron homeostasis including iron-regulatory proteins (e.g. ferritin and hepcidin)[15], as well as iron-related proteins with restricted tissue expression (e.g. TfR2-Transferrin Receptor 2, HFE-encoding Human hemochromatosis protein, and HAMP-hepcidin) [16]. As mentioned, iron is associated with inflammation in the setting of T2DM. More directly, iron overload has been demonstrated to promote adipocyte insulin resistance [7, 17]. Moreover, insulin treatment promotes iron uptake by increasing cell-surface expression of TfR1 (Transferrin Receptor 1) in adipocytes [18]. These studies, combined with the evidence that macrophages play a predominant role in controlling systemic iron recycling [19], raise the possibility that disrupted iron handling by adipose tissue macrophages (ATM) contributes to adipose tissue dysfunction. Furthermore, animal studies have demonstrated that obesity induces an increase in M1 polarization [20, 21], which, based on in vitro studies, may further promote iron deposition [22]. Interestingly, Orr et. al. identified a population of adipose tissue macrophage with an iron handling phenotype and indicated that high fat diet promotes iron partitioning to adipocytes and reduces the iron handling capacity of adipose tissue macrophage [23]. In addition, increasing amounts of evidence suggest that hypoxia can exert a profound impact on adipose tissue function. Hypoxia-sensing pathway manifested by hypoxia-inducible factor (HIF) 2α is associated with adipose tissue inflammation, glucose homeostasis, lipid metabolism, and production of adipokines and pro-inflammatory cytokines in adipose tissue [24, 25]. Studies have shown that HIF-2α directly regulates iron regulation (e.g. iron regulators DMT1 or divalent metal transporter 1 also known as solute carrier family [SLC] 11, member 2 [SLC11A2]), Dcytb (Duodenal cytochrome B), FTH (ferritin heavy chain) and FPN (ferroportin also known as SLC40A1))[26, 27] and inflammation (e.g. TNFα) [28]. However, the direct metabolic consequences of iron deposition are still unclear.

KK/HIJ (KK) mice have been used as a polygenic mouse model of obesity and insulin resistance. Interestingly, the serum iron level is more than 2-fold higher in this strain compared with more commonly used mouse strains such as C57BL/6J or C57BL/10J [29]. The combination of these factors suggests the KK/HIJ mouse to be a potentially useful model for iron and obesity-related research studies. In subsets of KK males, we have observed grossly evident adipose tissue remodeling characterized by iron deposition within the epididymal adipose depot. In our pilot study, we observed that approximately 50% of KK males have distinctly discolored epididymal adipose tissue depots. We determined that this was directly associated with a robust increase in iron concentration in this adipose depot. Furthermore, we noted that the remainder of the KK males had normal adipose tissue iron levels (in line with KK females and both genders of the C57BL/6J strain). This observation motivated us to further characterize this phenomenon in male KK/HIJ mice. To our knowledge, there have not been previous studies addressing this increased tissue iron phenotype.

In this study, we identified two distinct groups of KK males: a normal iron adipose tissue group (NI) and a high iron adipose tissue group (HI). Our aim was to characterize the iron dysregulation phenotype in the epididymal adipose tissue and evaluate adipose tissue inflammation and remodeling in this polygenic obese male mouse model.

## Materials and methods

### Animals and tissues

**Fig 1:**
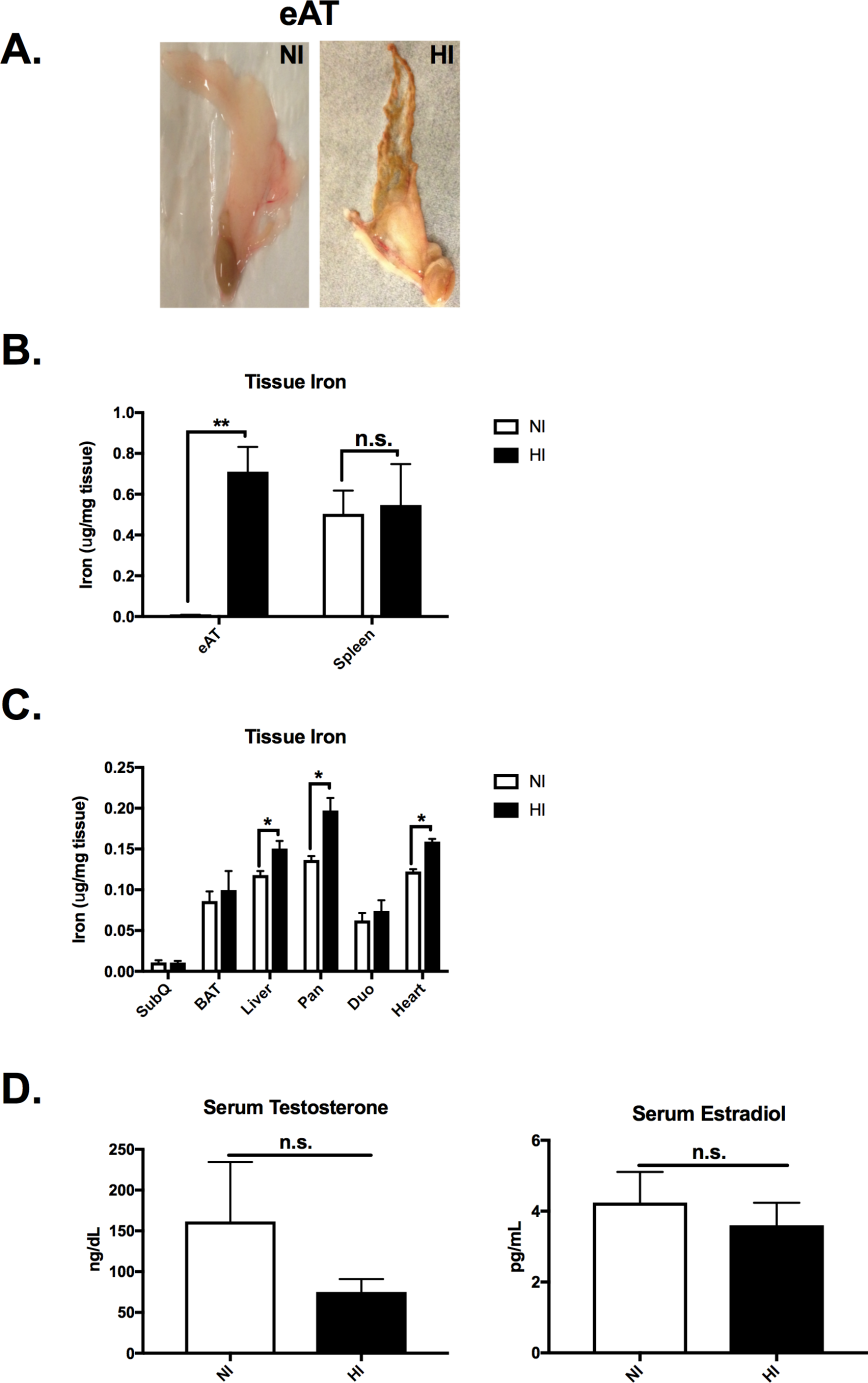
Iron deposition is tissue specific. A) Gross pictures of eAT from NI and HI groups. B) eAT iron was significantly elevated in HI group. C) Common iron deposition tissues such as liver, pancreas and heart had elevated iron levels in HI. However, iron deposition in duodenum or other adipose tissue depots (i.e. subcutaneous and brown adipose tissue) was not elevated in HI group. D) Serum testosterone and estradiol levels were not significantly different between NI and HI groups. *Abbreviations*: eAT, epididymal adipose tissue; SubQ, subcutaneous adipose tissue; BAT, brown adipose tissue; Pan, pancreas; Duo, duodenum. **p<0.01 NI (open bar) *vs* HI (filled bar).

Male KK/HIJ mice were obtained through in-house breeding at the University of Michigan from mice originally purchased from Jackson Laboratories (Strain #002106). Twenty-seven male KK mice aged 47–79 weeks were euthanized and checked for the presence of epididymal adipose tissue (eAT) discoloration (**Fig 1A** and subsequently eAT iron concentration). The mice with discolored eAT were assigned to “High Iron adipose tissue” group (HI, n = 14), while mice with normal colored eAT were assigned to “Normal Iron adipose tissue” group (NI, n = 13). HI group was associated with >100-fold higher iron concentration than NI. Five female KK mice (~ 34 weeks of age) from the same colony living under the same conditions were also euthanized for gonadal adipose tissue iron concentration. Ten male C57BL/6J mice at age of 8 weeks were randomly assigned to normal chow diet (NCD) or high fat diet (HFD) groups for 12-week diet intervention. Subcutaneous and epididymal adipose tissue samples from the NCD and HFD mice were collected. All mice were maintained in a temperature controlled environment under a standard 12 hrs light-dark cycle and provided *ad libitum* access to food and water throughout the study. At the end of the studies, animals were euthanized with CO_2_ in an appropriate chamber. The animal care and experimentation were overseen and approved by the University of Michigan Committee on Use and Care of Animals.

### Tissue iron assay

The liver, spleen, heart, and pancreas as well as epididymal, perirenal, and brown adipose tissue were weighed and harvested from each mouse. Gonadal adipose tissue samples were also harvested from the five KK female mice. Tissue non-heme iron concentration was determined using a commonly used, non-commercial iron chromogen colorimetric assay (main compositions including hydrochloric acid and trichloroacetic acid) described previously [30]. In brief, ~25mg tissues were homogenized with high-purity water (10 μl/1mg tissue). Equal volume of homogenized tissue (150 μl) and acid solution (1M HCl, 10% trichloroacetic) were mixed and then incubated in a 95°C heating block for 1hr. The samples were then vortexed and spun at 16000 X g for 10min at room temperature. For adipose tissue, the lipid layer was carefully removed and supernatant was transferred to a new tube to get rid of any lipid contamination. 50 μl sample aliquots or iron standards were mixed with 50 μl iron assay (1:1 ratio for 1mM Ferrozine and 3M Sodium Acetate, with 1% mercaptoacetic acid). After ~30 min, the absorbance was measured at 562 nm with Biotek Synergy2 plate reader (BioTek, Winooski, VT).

### Histology and immunofluorescence staining

eAT samples were fixed and stored in formalin after being harvested from the animals. Tissues then were transferred to histology cassettes individually and stored in 70% ethanol. The UM Histology Core performed paraffin processing, embedding, sectioning, and staining. The staining included hemotoxylin and eosin (H&E), F4/80, trichrome, Caspase3 and Perl’s Prussian Blue staining for eAT. Liver H&E staining was processed. Pancreas iron staining with enhanced Perl’s Prussian Blue staining (with 0.025% 3,3'-diaminobenzidine and 0.005% H_2_O_2_) was also performed. The histology was quantified using ImageJ V1.51h software developed by Wayne Rasband from National Institutes of Health. For the immunofluorescence staining, the paraffin sections were rehydrated, performed antigen retrieval and analyzed as described previously [28]. Primary antibodies against HIF-2α from Novus Biologicals (Littleton, CO) and anti-Nitro Tyrosine from Abcam (Cambridge, MA) were used.

### Western blot analysis

Western blot analyses were performed as previously described (44). Antibodies including T-FTH, AKT, p-AKT were from Cell Signaling Technology Inc. (Danvers, MA); DMT1 and FPN antibodies were from Alpha Diagnostic Intl. Inc. (San Antonio, Texas), TfR antibody was from Thermo Fisher Scientific Inc. (Grand Island, NY). DcytB was from Novus Biologicals (Littleton, CO), and GAPDH was from Santa Cruz Biotechnology (Santa Cruz, CA). The western blot results were quantified using ImageJ V1.51h software developed by Wayne Rasband from National Institutes of Health.

### Serum measurements

Intraperitoneal glucose tolerance tests (GTT) were performed in NI and HI group (a cohort of 8 KK male mice, NI: n = 4, age = 14.2 ± 0.37 months old; HI: n = 4, age = 14.4 ± 0.41 months old;). In each case, mice were fasted for 5 hrs (0800–1300) and were subsequently injected with glucose (1.5 g/kg body weight. i.p.). Tail blood was collected at 0, 30, 60, 90 and 120 min. Blood glucose concentrations were measured using a commercially available glucometer (Abbott Laboratories, Abbott Park, IL). Following a 5 hrs fasting, serum insulin, adiponectin and leptin were measured using commercially available ELISA kits (Crystal Chem, Downers Grove, IL) according to manufacturer’s instructions. Serum testosterone and estradiol were measured by The University of Virginia center for Research in Reproduction Ligand Assay and Analysis Core. Serum iron was analyzed using the QuantiChrom iron assay kit (Bioassay Systems, Hayward, CA) following the manufacturer's protocol. Insulin resistance was indicated by the homeostasis model assessment–estimated insulin resistance (HOMA-IR) index, which was calculated according to the following equation:

$$HOMA-IR=\frac{Fasting\;Glucose(mg/dl)\times Fasting\;Insulin\;(\mu U/mL)}{405}$$

### Gene expression qRT-PCR

Total RNA was extracted from eAT and liver using the RNAqueous kit (Life Technologies, Grand Island, NY) according to the manufacturer’s instructions. cDNA was synthesized using High Capacity cDNA Reverse Transcription Kit (Applied Bio systems, CA). Real-time PCR was used to amplify the cDNA with gene specific primers using Taqman gene expression assay (Applied Biosystems, FosterCity, CA) for *Tnfα* and *Lep* (Leptin) gene expression. Fast SYBR green Master Mix (Applied Biosystems, Grand Island, NY) was used for additional gene expression studies (**Table 1**). Real-time PCR was carried out by using StepOne plus software (Applied Biosystems, Foster City, CA). Results then were analyzed using the 2^-ΔΔCT^ method described previously [31, 32].

**Table 1:** Primers sequences for gene expression.

Gene	Forward (5’-->3’)	Reverse (5’-->3’)
*Gapdh*	TGAAGCAGGCATCTGAGGG	CGAAGGTGGAAGAGTGGGAG
*Hamp*	CCTGAGCAGCACCACCTATCT	GCTTTCTTCCCCGTGCAAAGG
*Slc11a2*	TTGGCAATCATTGGTTCTGA	CTTCCGCAAGCCATATTTGT
*Slc40a1*	ATGGGAACTGTGGCCTTCAC	TCCAGGCATGAATACGGAGA
*Vegf*	CCACGTCAGAGAGCAACATCA	TCATTCTCTCTATGTGCTGGCTTT
*Il10*	GCTCTTACTGACTGGCATGAG	CGCAGCTCTAGGAGCATGTG
*Dcytb*	CATCCTCGCCATCATCTC	GGCATTGCCTCCATTTAGCTG
*TNFa*	AGGGTCTGGGCCATAGAACT	CCACCACGCTCTTCTGTCTAC
*Slc11a2-IRE*	TGTTTGATTGCATTGGGTCTG	CGCTCAGCAGGACTTTCGAG
*TfR1*	GGAAGACTCTGCTTTGCAGCTAT	GCCCAGGTAGCCCATCATGA
*Hif-2a*	TGAGTTGGCTCATGAGTTGC	TATGTGTCCGAAGGAAGCTG
*Ccl2*	ATTGGGATCATCTTGCTGGT	CCTGCTGTTCACAGTTGCC
*Cxcl1*	TCTCCGTTACTTGGGGACAC	CCACACTCAAGAATGGTCGC
*Adipoq*	GGAGATGCAGGTCTTCTTGG	ATGTTGCAGTAGAACTTGCC
*Lep*	TGAAGCCCAGGAATGAAGTC	TCAAGACCATTGTCACCAGG

*Abbreviations*: *Hamp*, hepcidin; *Slc11a2*, DMT1; *Slc40a1*, ferroportin; *Vegf*, Vascular endothelial growth factor; *Il10*, interlukin 10; *Dcytb*, Duodenum cytochome B; *TNFα* tumor necrosis factorα; *TfR1*, transferrin receptor 1; *HIF-2α*, Hypoxia inducible factor 2*α*; *Adipoq*, adiponection; *Lep*, leptin.

### Statistics

The primary comparison in this study was between the NI and HI groups as well as NCD and HFD groups, so an independent student’s t-test was applied to determine significant differences between the groups. To evaluate the association between eAT iron and adipose tissue inflammation markers, Pearson’s correlation tests were performed. Results were expressed as Mean (±SEM). The statistical analysis was carried out using the SPSS statistics package (IBM SPSS statistics 21). P-values less than 0.05 were considered statistically significant.

## Results

### Iron deposition is tissue and adipose depot-specific

The eAT had a bronzing characteristic that reflects (**Fig 1A**) iron concentrations in eAT, which were 115- fold higher in HI compared NI (NI: 0.0062 ± 0.002 μg/mg tissue; HI: 0.71 ± 0.12 μg/mg tissue; p<0.01, **Fig 1B**). The gonadal adipose tissue (gAT) iron concentration in female KK mice was similar to eAT iron from the male NI group (gAT: 0.0082± 0.001 μg/mg tissue, p>0.05). In addition, the liver (27%, p<0.01), pancreas (44%, p<0.01) and heart (30%, p<0.01) had significantly higher iron concentration in HI compared with NI (**Fig 1C**). Consistent with tissue iron assay, enhanced Pearl’s Prussian Blue staining in the pancreas also showed a significantly greater iron deposition. Moreover, islet iron is mainly in the beta cells in the NI group, whereas there was a robust increase in iron level in both alpha and beta cells in the HI group (**S1A Fig**). However, other adipose tissue depots, such as subcutaneous and brown adipose tissue, did not have significant differences in iron concentration between the two groups (p>0.05, **Fig 1C**). Duodenum, as the main dietary iron absorption site, was also not different between NI and HI (p>0.05, **Fig 1C**). Serum levels of testosterone and the most prevalent form of estrogen, estradiol, were also evaluated, no difference between the NI and HI groups was observed (p<0.01, **Fig 1D**). To test if the iron deposition in KK male is strain specific, we measured iron levels in eAT and subcutaneous adipose tissue from 12-week NCD or HFD intervention male mice. Both eAT and subcutaneous adipose tissue iron levels were not significantly different between NCD and HFD (p>0.05, **S1B Fig**). Therefore, in addition to the major iron deposition organs including liver, pancreas and heart, iron overload in adipose tissue was specific to eAT in the HI group in the KK male mice without dietary challenge and was not evident by 12-week diet induced obesity in C57BL/6J.

### Iron deposition is associated with adipose tissue remodeling

**Fig 2:**
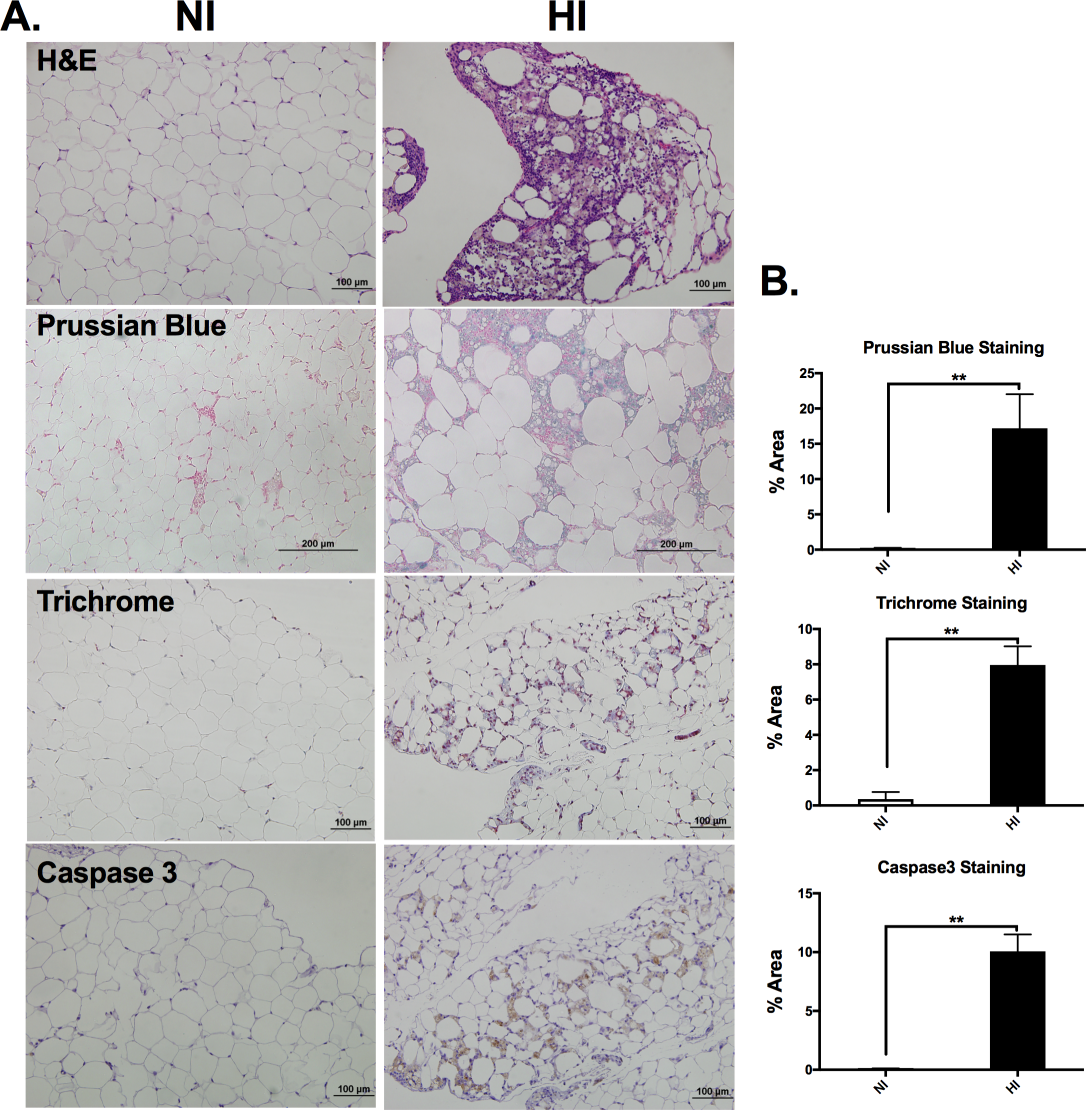
A robust tissue remodeling in the HI mice epididymal fat pads. **A)** Representitive adipose tissue histology from both NI (left) and HI (right) groups. H&E staining revealed robust adipose tissue cellularity changes in the HI group. A high number of non-adipocytes were observed residing between the adipocytes, Prussian blue staining also showed a robust iron overload in the eAT of HI group. Masson's trichrome stain revealed a significant amount of collagen fibers present among adipocytes in HI compared with NI. Caspase 3 staining suggested greater apoptosis in the HI group. B) Quantification for Prussian blue, Thricrome and caspase 3 staining. *Colors*: F4/80, brown; Trichrome, blue; Caspase 3, brown; Prussian blue, blue. Scale bars represent 100μm. **p<0.01 NI (open bar) *vs* HI (filled bar).

Tissue weight indicated that epididymal adipose tissue and liver were smaller in HI compared with NI group (p<0.05, **Table 2**), while the heart and relative spleen weights were not different between the two groups (p>0.05, **Table 2**). Histology data with H&E staining revealed robust adipose tissue cellularity changes in the HI group. The individual adipocyte size was in a much wider range and was irregular in HI compared with NI. In addition, a high number of stromal vascular cells were observed residing between the adipocytes (**Fig 2A**). Perl’s Prussian blue staining showed robust iron retention in the adipocytes as well as stromal vascular cells infiltrated to the adipose tissue (**Fig 2A & 2B**). By using Masson's trichrome stain for collagen fibers, we observed a significant amount of collagen fiber staining present among adipocytes in HI compared with NI (**Fig 2A & 2B**). In addition, Caspase 3 staining suggests greater apoptosis in eAT of the HI group (**Fig 2A & 2B**). Our data suggested that the high iron deposition in the eAT is associated with dramatic adipose tissue remodeling including eAT morphologic changes, increased fibrosis and apoptosis.

**Table 2:** Mouse tissue weights.

Tissue weight	NI (n = 6)	HI (n = 4)	*P values*
Body Weight (g)	31.03 ± 1.19	28.98 ± 6.14	0.267
Epididymal AT (g)	0.62 ± 0.12	0.14 ± 0.02	0.026*
%Epididymal AT	1.95 ± 0.33	0.50 ± 0.05	0.019*
Perirenal AT (g)	0.45 ± 0.10	0.25 ± 0.04	0.344
%Perirenal AT	1.4 ± 0.30	0.90 ± 0.09	0.389
Heart (g)	0.15 ± 0.01	0.14 ± 0.00	0.586
% Heart	0.47 ± 0.02	0.50 ± 0.02	0.606
Liver (g)	1.45 ± 0.08	1.17 ± 0.02	0.035*
% Liver	4.73 ±0.38	4.05 ± 0.11	0.201
Spleen (g)	0.12 ± 0.01	0.08 ± 0.00	0.034*
% Spleen	0.39 ± 0.03	0.28 ±0.03	0.064
Pancreas (g)	0.21 ± 0.03	0.24 ± 0.03	0.588

*Abbreviations*: AT, adipose tissue. Data are presented as mean ± SE

* p<0.05 NI *v*.*s*. HI

### The local eAT iron change does not result in a systemic change

**Fig 3:**
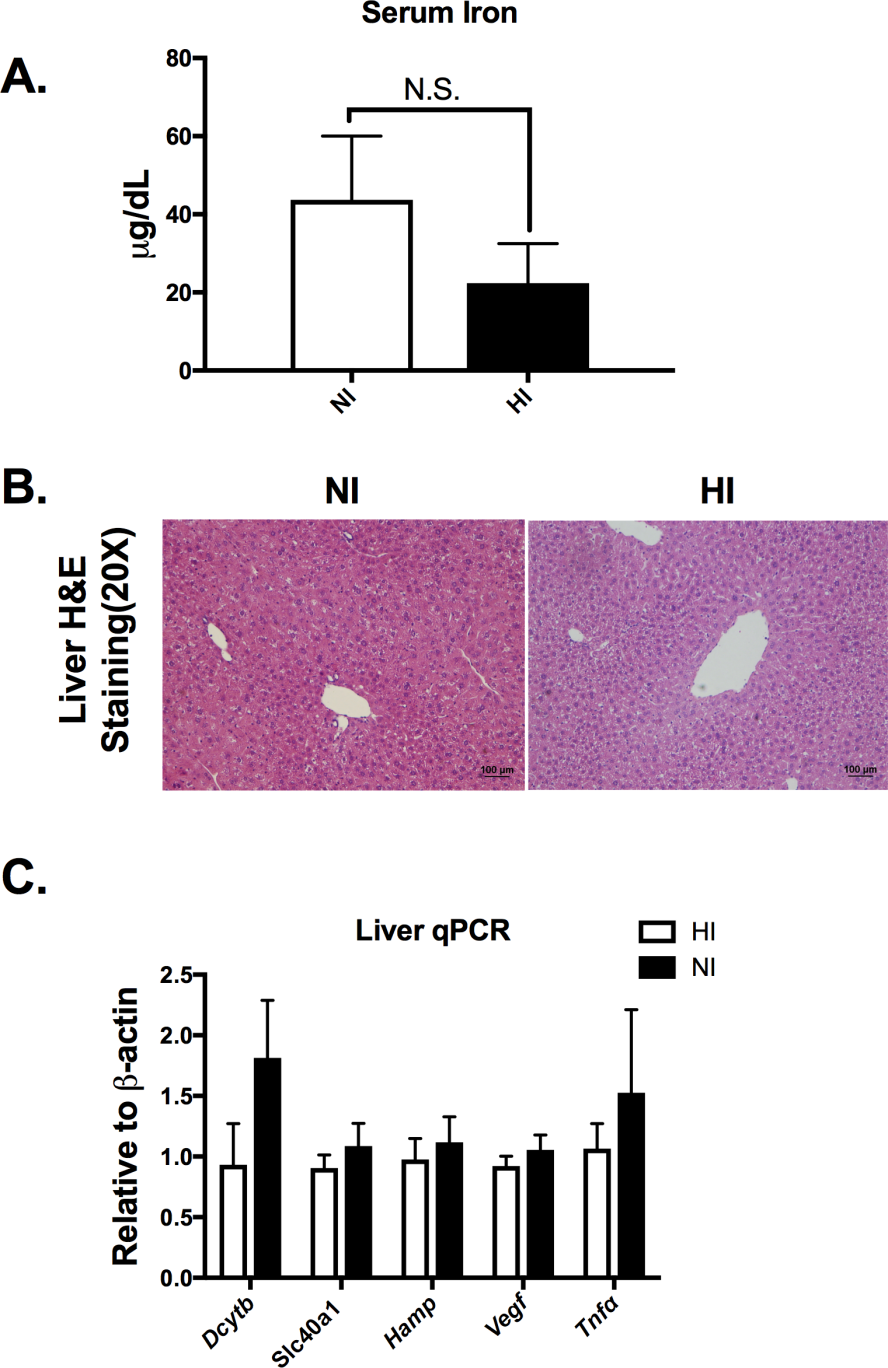
No systemic change despite local eAT remodeling and iron change. A) Serum iron levels between the NI and HI groups. B) Representative H&E staining for liver in NI and HI groups. C) Liver iron-related and inflammatory gene expression. *Abbreviations*: *DcytB*, Duodenal cytochrome b; *Fpn*, ferroportin; *Hamp*, hepcidin; *Vegfa*, Vascular endothelial growth factor A; *Tnf*α, tumor necrosis factor; NI (open bar, n = 6) *v*.*s*. HI (filled bar, n = 6). *p< 0.05.

Serum iron was not different (NI: 43.71 ± 16.32 μg/dL; HI: 47.78 ± 26.44 μg/dL, p>0.05, **Fig 3A**), despite differences in tissue iron deposition between the groups. Liver H&E staining did not reveal a significant histological difference between NI and HI (**Fig 3B**). In addition, expression of liver iron-related (*Dcytb*, *Slc40a1*, and Hamp) and inflammatory (*Tnf* α, tumor necrosis factor) genes was not different between HI and NI group (p>0.05, **Fig 3C**). This indicates that the profound local iron change in eAT had not induced a systemic change, at least at the time of our observation.

### Elevated iron deposition in the eAT is associated with increased HIF-2α

**Fig 4:**
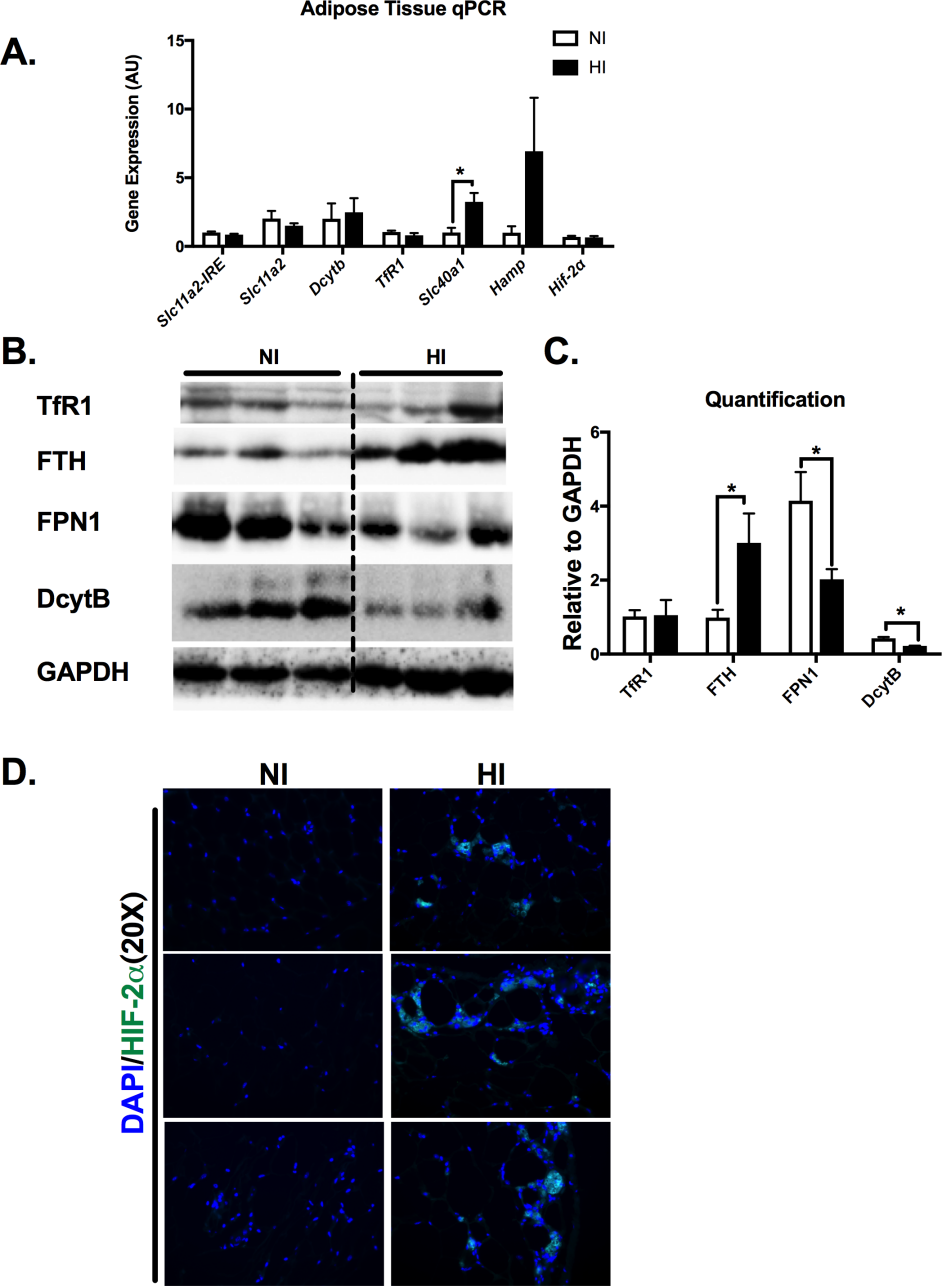
The elevated iron deposition in the eAT is associated with increased HIF-2α accumulation. **A)** eAT gene expression with iron-regulating gene markers. B)&C) adipose tissue iron-regulating protein levels and the quantifaction after normalizing with GAPDH. D) immunoflorescent staining for HIF-2α in NI and HI eAT. *Abbreviations*: DMT1-IRE, Divalent metal transporter 1-Iron response element, *DcytB*, Duodenal cytochrome b; TfR1, *Fpn*, ferroportin; *Hamp*, hepcidin; Hif-2α, hypoxia inducible factor 2α. *p< 0.05.

No statistical difference was evident between the groups for the gene expression of *Slc11a2* (solute carrier family 11 member 2, the gene encodes the cellular iron intake marker divalent metal transporter 1, DMT1), *Dcytb* (encoding iron-regulated protein duodenum Cytochrome B Reductase 1) or *Hamp* (encoding hepcidin, which degrades ferroportin) (p>0.05, **Fig 4A**). However, the gene expression of *Slc40a1* (encoding iron exporter protein ferroportin *(FPN1)*) was significantly higher (~3-fold) in the eAT of HI compared with NI (p<0.05, **Fig 4A).** In addition, iron transporters including TfR1, FPN and DMT1 were not changed, while FTH was increased and DcytB was decreased in HI group (**Fig 4B & 4C**). Nitrotyrosine, an oxidative stress marker, was significantly elevated in the HI eAT as well as in tissues with increased iron deposition tissues such as pancreas and liver (**S2 Fig**). These indicate that elevated iron levels are associated with increased oxidative stress in these tissues. To further investigate the cause of increasing iron levels and inflammation in eAT, HIF-2α immunofluorescence staining showed a significant increase of HIF-2α in the HI eAT nucleus (**Fig 4D**), indicating that HIF-2α might be contributing to the elevated iron and inflammation.

### Iron deposition is associated with increased adipose tissue inflammation

**Fig 5:**
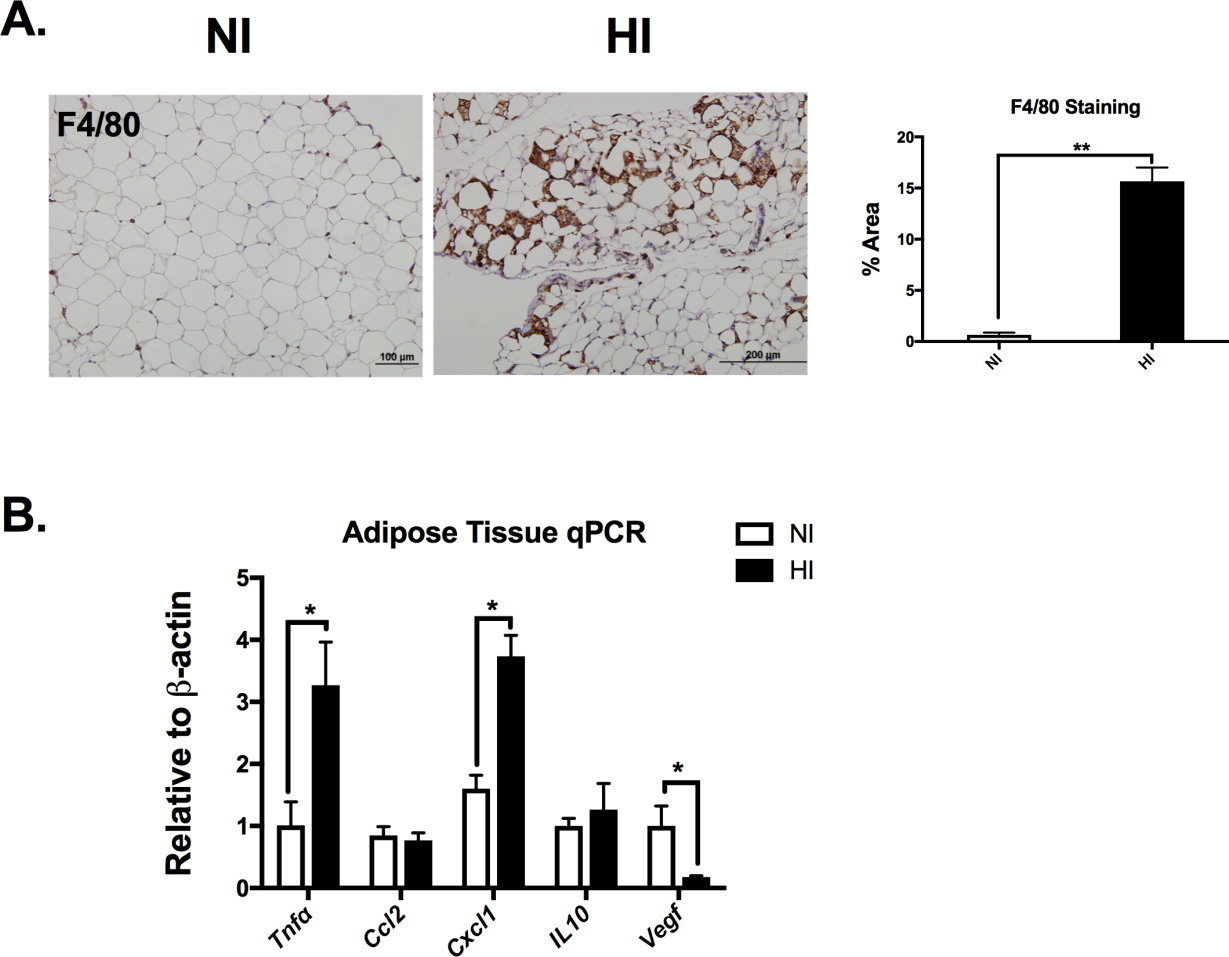
Iron deposition is associated with increased adipose tissue inflammation. **A)** Representative adipose tissue F4/80 inmmunoflorescent staining from both NI (left) and HI (right) groups. Quantification for F4/80 antibody staining revealed macrophage clustering among the adipocytes in HI group. B) eAT gene expression with inflammatory gene markers. *Abbreviations*: *Tnf*α, tumor necrosis factor; *Ccl2*, C-C Motif Chemokine Ligand 2; *Cxcl1*, C-X-C Motif Chemokine Ligand 1, *Il10*, interlukin 10; *Vegf*α, Vascular endothelial growth factor A. *p< 0.05.

To characterize the adipose tissue inflammation in the eAT, F4/80 antibody staining was used and revealed macrophage clustering among the adipocytes (**Fig 5A**). Gene expression studies of eAT were used to examine differences in genes relevant to adipose tissue inflammation and iron regulation between NI and HI. The pro-inflammatory gene marker *Tnfα* and *Cxcl1* (encodes C-X-C Motif Chemokine Ligand 1, an inflammatory protein that acts in part as a chemoattractant) were significantly higher in eAT of HI group compared with NI group (p<0.01, **Fig 5B**), while the anti-inflammatory gene marker *Il10* was not different in HI and NI. Gene expression of *Vegfa* (Vascular endothelial growth factor A) was on average 82% lower in the HI compared to NI, but this difference did not reach statistical significance (p = 0.058).

### Local but not systemic glucose homeostasis was impaired with increased adipose tissue iron accumulation

**Fig 6:**
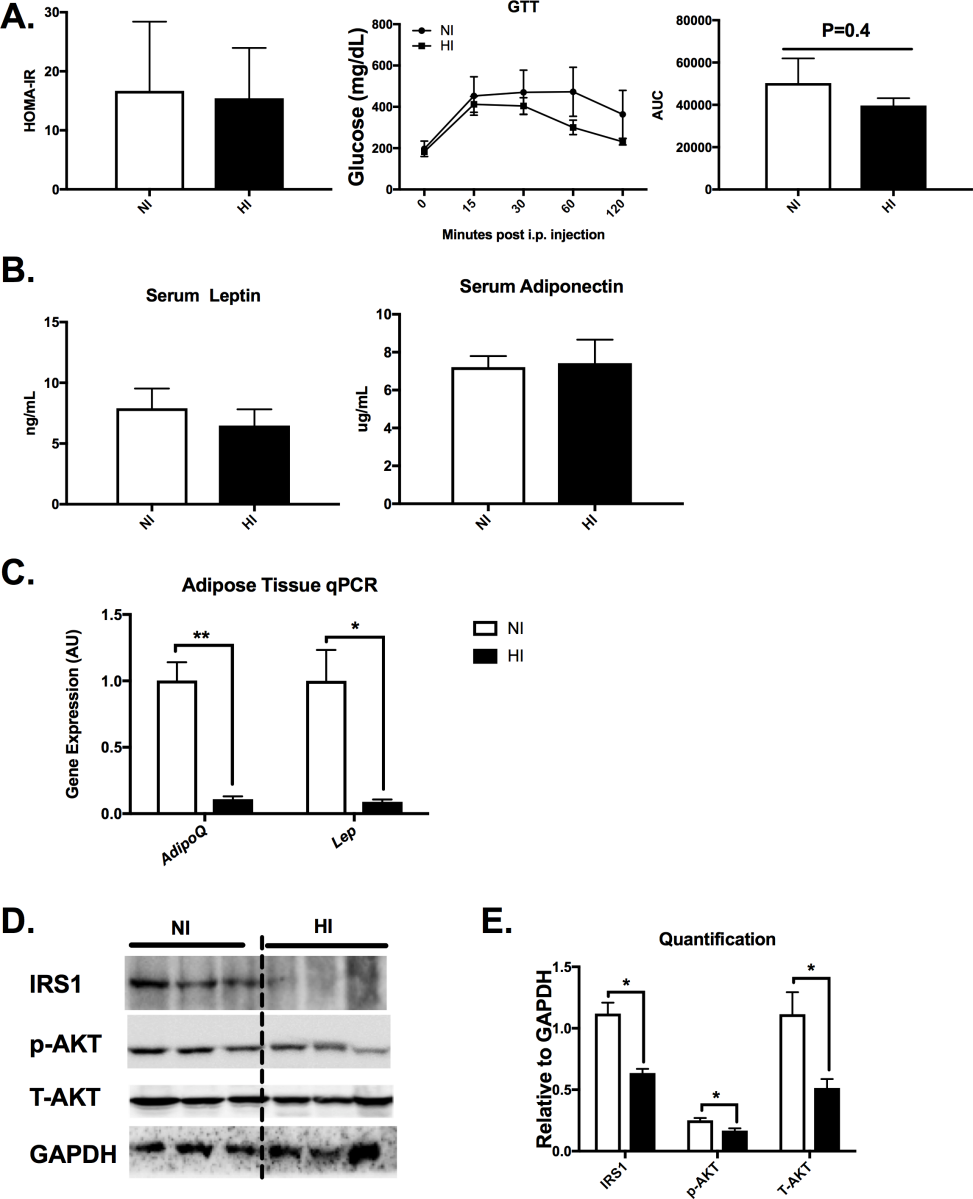
Local glucose homeostasis was impaired with increased adipose tissue iron accumulation. A) Insulin resistance index HOMA-IR, glucose tolerance test (GTT) and the area under the curve (AUC) in both NI and HI groups. B)Serum adiponectin and leptin in NI (n = 6) and HI (n = 4) mice. C) adipose tissiue gene expression with adipokine markers in NI and HI groups. D&E) adipose tissue insulin signaling protein levels and the quantification after normalizing with GAPDH. *p< 0.05.

The analysis of metabolic markers including glucose tolerance test (GTT), area under the curve(AUC), serum glucose (NI: 103.48 ±13.69 μg/dL; HI: 90.37 ± 24.83 μg/dL, p>0.65), serum insulin (NI: 1.83± 1.16 ng/mL; HI: 2.49 ± 1.13 ng/mL, p>0.05) and HOMA-IR (p>0.05, **Fig 6A**) were not different between HI and NI groups. Serum adipokines that are associated with adiposity and insulin sensitivity (e.g. serum adiponectin and serum leptin) were also investigated. Neither serum adiponectin (HI: 7.21 ± 0.58 μg/mL; NI: 7.42 ± 1.25 μg/mL, p>0.05) nor serum leptin (NI: 7.91 ± 1.62 ng/ml; HI: 6.48 ± 1.34 ng/ml, p>0.05) were significantly different between the HI and NI groups (p>0.05, **Fig 6B**). However, consistent with the robust remodeling of eAT in the HI group, the gene expression of leptin and adiponectin in the eAT was markedly reduced compared with eAT of NI group (p<0.01, **Fig 6C**). Decreased insulin signaling, evaluated by the insulin receptor substrate (IRS1) protein level (**Fig 6D and 6E**), was down-regulated in the HI mice. Protein kinase B (AKT) was also assessed. Although no significant difference was evident for the p-AKT/T-AKT ratio, a total decrease of p-AKT and total AKT expression was observed (**Fig 6D and 6E**). Overall, iron deposition was localized to specific tissues and was observed to produce overt differences in local but not systemic insulin signaling impairment between NI and HI mice.

## Discussion

In our study, we have characterized a polygenic obese mouse model with a propensity for tissue iron deposition in the epididymal adipose depot. We found that the adipose tissue deposition of iron in a subset of male KK/HIJ mice was specific to the eAT depot and not evident in the subcutaneous or brown adipose tissue depots. Moreover, these same animals had evidence of greater iron deposition in traditional tissues of iron overload including the liver, heart, and pancreas. The increased tissue iron was also accompanied with elevated oxidative stress in eAT, liver and pancreas, suggesting an interesting link among iron, oxidative stress and tissue remodeling in at least eAT. The increase in eAT macrophages, collagen and cell death suggests robust tissue remodeling in the high iron subgroup. These alterations in iron regulation and inflammation in the eAT might be due to accumulated HIF-2α, which could result in impaired local insulin signaling. Nevertheless, no systemic alterations were observed in mice with this localized eAT iron deposition including markers of glucose homeostasis and the serum concentration of the adipocytokines, leptin and adiponectin.

Analysis of the adipose tissue revealed a robust remodeling of the eAT in the HI group. In addition to the significantly increased eAT iron, our studies revealed an increase of macrophages (F4/80 staining) among the adipocytes as well as higher gene expression of the inflammatory marker, *Tnfα*. As the macrophage is among the most important cell types for iron storage, it is tempting to speculate that macrophage infiltration may have preceded the elevation of iron. HIF-2α plays a critical role in maintaining iron balance and governing inflammation. It has been well-studied that HIF-2α directly regulates the transcription of numerous genes involved in iron regulation, inflammation, and angiogenesis [27, 28]. We hypothesize that HIF-2α might play a role in the KK male mice regarding the strong iron phenotype. Interestingly, a robust increase in HIF-2α was observed in the HI eAT, which might provide insight to the cause of the adipose tissue alteration. HIF-2α direct target genes include iron regulators such as *Slc11a2*, *Dcytb*, *Slc40a1* as well as inflammatory cytokines such as *Tnfα*. Indeed, we observed increased *Slc40a1* and *Tnfα* gene expression as well as increased ferritin protein level. Based on the adipose tissue phenotype, especially the elevated iron deposition, it is possible that the highly increased iron in the eAT exerted a negative feedback, which would shut down the iron uptake pathways. This might be why we observed no change of TfR and even a reduction of DMT1 and DcytB protein level in the eAT. Therefore, it is possible that the increased adiposity led to a hypoxic environment in the eAT of a subset of KK male mice, which led to the observed increase in eAT iron, macrophage accumulation, and inflammation. Other observed phenotypes include increased cell death (caspase 3 staining) and fibrosis (trichrome staining) in the eAT. Several published studies have suggested that adipocyte death and increased fibrosis can promote adipose tissue macrophage infiltration. For example, Cinti et al. showed that adipocyte necrosis is a significant phagocytic stimulus that promotes ATM infiltration [33]. Adipose tissue fibrosis, however, is associated with reduced plasticity and is considered a key indication of metabolic dysfunction [34]. Overall, our phenotype was very consistent with the phenotype observed by Dongiovanni et al. following a high iron diet intervention in mice. For example, they observed a robust remodeling of the eAT characterized by a reduced tissue weight, a significant (~10-fold) increase in adipose tissue iron, a decrease in *leptin* expression and an elevation in eAT *Hamp* compared to control diet mice [35], consistent with the alterations we observed specifically in the eAT between the HI and NI mice. An increase in liver iron concentration without a detectable change in liver inflammation or histology was also observed in both studies. Oxidative stress has been considered as one of the deleterious factors leading to insulin resistance. Interestingly, we observed an increased oxidative stress in iron deposition tissues including eAT, liver and pancreas in HI group mice. In contrast to this high iron diet intervention, however, we did not observe differences in serum iron or circulating adiponectin concentration. Although we did observe a local disruption of normal adipose tissue gene expression including *Lep* (leptin), *Adipoq* (adiponectin) and *Vegfa* (vascular endothelial cell growth factor A), we did not observe systemic changes of the protein levels. Taken together, our results suggest that HI group mice had evidence of localized adipose tissue remodeling in the eAT similar to those previously observed in mice receiving a high iron diet. However, additional studies are needed to further our understanding of the direct cause of iron accumulation and adipose tissue remodeling observed in the current study.

The robust local changes in eAT of the HI group significantly impaired the local insulin signaling but did not exacerbate the systemic metabolic status in KK male mice. Studies have shown that iron is negatively associated with adiponectin transcription and that loss of the adipocyte iron export channel, ferroportin, can result in adipocyte iron accumulation, decreased adiponectin, and insulin resistance in mice [7, 36]. Consistent with previous studies [7, 23], we observed that eAT iron is negatively correlated with adiponectin gene expression in the epididymal adipose tissue (r^2^ = 0.78). However, we did not observe a significant difference in circulating adiponectin between the HI and NI groups or a strong correlation between eAT iron and circulating (serum) adiponectin. Furthermore, unlike other studies showing that iron overload impairs glucose homeostasis and iron depletion improves insulin sensitivity [7, 14, 35], in our study, the analysis of GTT as well as fasting glucose and the insulin resistance index, HOMA-IR, revealed no difference between the HI and NI groups. We hypothesized that the iron deposition in the KK adipose tissue is associated with local insulin resistance in the adipose tissue rather than systemic metabolic alterations. Indeed, we observed the local IRS1 protein level was abolished and the gene expression of Socs 3 was elevated in the HI group. Therefore, the effect of eAT iron deposition is local rather than whole body. Our histology supports this notion as adipocyte density appears considerably reduced and inflammatory cells are highly prevalent in the remodeled HI eAT.

To our knowledge, this is the first study demonstrating that about 50% of KK male mice are predisposed to iron accumulation and remodeling specifically in the eAT depot. Notably, these animals had a significant elevation of tissue iron in liver, pancreas and heart (27–44%, p<0.01) compared with the NI group, suggesting that there was a systemic iron overload in tissues sensitive to iron deposition. However, no elevation of iron levels was evident in subcutaneous or brown adipose tissue depots indicating that the eAT may have a unique milieu regarding iron deposition. In addition, female KK mice were also evaluated for evidence of iron deposition in adipose tissue, but levels were similar to NI eAT iron and consistently low in the gonadal adipose depot. This gender- and tissue-specific iron deposition represents an interesting phenomenon that may have important clues to our understanding of the prevalence of iron toxicity and iron deposition in adipose tissue, in particular. This eAT tissue-specificity is consistent with other studies, where high iron diet or 24-week high fat diet-induced obese C57BL/6J showed a tissue iron increase in only the epididymal AT depot [35, 37]. In addition, after 12-week high fat diet intervention, C57BL/6J mice did not have significant different in eAT and subcutaneous adipose tissue iron levels compared with the normal chow diet counterpart. Our finding is consistent with other study [37] and indicates the adipose tissue iron phenotype requires longer than 12-week high fat diet exposure. Due to the male specificity in eAT iron deposition, serum testosterone and estradiol (the most prevalent form of estrogen) levels were also evaluated. However, our data did not reveal a systemic testosterone or estradiol levels difference between the NI and HI groups. The unique alterations of this specific adipose tissue depot may relate to variances in sympathetic innervation or other localized environmental differences. One considerable difference previously documented between the milieu of the eAT and other adipose depots is the presence of high levels of estrogen sulfotransferase (EST) in the eAT [38]. This enzyme promotes the sulfoconjugation of estrogen. High levels of EST in eAT can lead to the rapid inactivation of estrogen locally and may relate to this gender- and tissue-specific iron deposition in male KK mice. Consistent with this concept, increased adipose tissue iron has been observed in female mice following ovariectomy [39]. In addition, several publications have also demonstrated that EST deficiency can increase epididymal adipose tissue mass and adipocyte size; whereas EST overexpression decreased primary adipocyte differentiation and induced a similar adipose tissue remodeling (e.g. significantly decreased adipose tissue mass) as in our current study [38, 40]. Given the remarkable parallels between our observations and these findings, we speculate that the tissue- and gender-specific iron accumulation in the KK male mice may result from an elevation of eAT estrogen inactivation by estrogen sulfotransferase.

Further study with iron manipulation (e.g. low iron diet intervention) in KK male mice will be necessary to improve our understanding of the cause of iron accumulation in eAT and systemic consequences of this condition. In addition, more studies are necessary to investigate the mechanism of HIF-2a accumulation and if it is essential to the phenotypic alterations we observed. However, the main focus of the current study was to better characterize the impact of the iron deposition on adipose tissue structure and function.

In conclusion, our study characterized the adipose tissue change and glucose homeostasis in KK mice with a grossly evident epididymal adipose tissue iron deposition. As the study of the male epididymal adipose depot is a commonly evaluated tissue, it is important to note that this depot may be unique regarding inflammation and iron deposition. We have observed in the KK strain a propensity for a site-specific iron accumulation in eAT that is associated with robust adipose tissue inflammation and remodeling. We speculate that the epididymal adipose tissue depot in this mouse strain has a special propensity for iron deposition in the setting of iron overload. This robust phenotype might be caused by the accumulation of hypoxia inducible factor HIF-2α within the eAT. Consequently, the increased local iron accumulation and local inflammation causes a local impairment of local insulin sensitivity. However, further studies are needed to characterize the systemic and local metabolic dysfunction as well as to identify the mechanism of the site-specific iron overload in the eAT. Overall, we propose that the KK mouse provides a robust model for examining the role of elevated iron in the setting of polygenic obesity.

## Supporting information

S1 Fig Pancreas iron staining and HFD intervention adipose tissue iron levels. A) Perl’s Prussian Blue staining in the pancreas from HI and NI groups. Top panel represents pancreas from NI and HI groups with 10X magnification, and lower panel is with 20X magnification. B) Tissue iron levels in eAT and subcutaneous adipose tissue from 12-weeks NCD or HFD intervention C57BL/6J male mice. NCD (open bar) *vs* HFD (filled bar). *Colors*: iron, brown.
(PDF)

S2 Fig Iron accumulation is associated with increased oxidative stress. Immunoflorescent staining for Nitrotyrosine eAT, liver and pancreas in NI and HI groups.
(PDF)

## References

[1] Jiang R, Manson JE, Meigs JB, Ma J, Rifai N, Hu FB. Body iron stores in relation to risk of type 2 diabetes in apparently healthy women. Jama 2004;291(6):711–7. doi: 10.1001/jama.291.6.711 14871914 10.1001/jama.291.6.711

[2] Forouhi NG, Harding AH, Allison M, Sandhu MS, Welch A, Luben R, et al Elevated serum ferritin levels predict new-onset type 2 diabetes: results from the EPIC-Norfolk prospective study. Diabetologia 2007;50(5):949–56. doi: 10.1007/s00125-007-0604-5 17333112 10.1007/s00125-007-0604-5

[3] Fernandez-Real JM, Penarroja G, Castro A, Garcia-Bragado F, Hernandez-Aguado I, Ricart W. Blood letting in high-ferritin type 2 diabetes: effects on insulin sensitivity and beta-cell function. Diabetes 2002;51(4):1000–4. 11916918 10.2337/diabetes.51.4.1000

[4] Cighetti G, Duca L, Bortone L, Sala S, Nava I, Fiorelli G, et al Oxidative status and malondialdehyde in beta-thalassaemia patients. European journal of clinical investigation 2002;32 Suppl 1:55–60. 11886433 10.1046/j.1365-2362.2002.0320s1055.x

[5] Walter PB, Fung EB, Killilea DW, Jiang Q, Hudes M, Madden J, et al Oxidative stress and inflammation in iron-overloaded patients with beta-thalassaemia or sickle cell disease. British journal of haematology 2006;135(2):254–63. doi: 10.1111/j.1365-2141.2006.06277.x 17010049 10.1111/j.1365-2141.2006.06277.x PMC2185791

[6] Tajima S, Ikeda Y, Sawada K, Yamano N, Horinouchi Y, Kihira Y, et al Iron reduction by deferoxamine leads to amelioration of adiposity via the regulation of oxidative stress and inflammation in obese and type 2 diabetes KKAy mice. American journal of physiology Endocrinology and metabolism 2012;302(1):E77–86. doi: 10.1152/ajpendo.00033.2011 21917632 10.1152/ajpendo.00033.2011

[7] Gabrielsen JS, Gao Y, Simcox JA, Huang J, Thorup D, Jones D, et al Adipocyte iron regulates adiponectin and insulin sensitivity. The Journal of clinical investigation 2012;122(10):3529–40. doi: 10.1172/JCI44421 22996660 10.1172/JCI44421 PMC3461897

[8] Hotamisligil GS. Inflammatory pathways and insulin action. International journal of obesity and related metabolic disorders: journal of the International Association for the Study of Obesity 2003;27 Suppl 3:S53–5. 10.1038/sj.ijo.0802502 14704746

[9] Hotamisligil GS, Arner P, Caro JF, Atkinson RL, Spiegelman BM. Increased adipose tissue expression of tumor necrosis factor-alpha in human obesity and insulin resistance. The Journal of clinical investigation 1995;95(5):2409–15. doi: 10.1172/JCI117936 7738205 10.1172/JCI117936 PMC295872

[10] Weisberg SP, McCann D, Desai M, Rosenbaum M, Leibel RL, Ferrante AW Jr. Obesity is associated with macrophage accumulation in adipose tissue. The Journal of clinical investigation 2003;112(12):1796–808. doi: 10.1172/JCI19246 14679176 10.1172/JCI19246 PMC296995

[11] Xu H, Barnes GT, Yang Q, Tan G, Yang D, Chou CJ, et al Chronic inflammation in fat plays a crucial role in the development of obesity-related insulin resistance. The Journal of clinical investigation 2003;112(12):1821–30. doi: 10.1172/JCI19451 14679177 10.1172/JCI19451 PMC296998

[12] Minamiyama Y, Takemura S, Kodai S, Shinkawa H, Tsukioka T, Ichikawa H, et al Iron restriction improves type 2 diabetes mellitus in Otsuka Long-Evans Tokushima fatty rats. American journal of physiology Endocrinology and metabolism 2010;298(6):E1140–9. doi: 10.1152/ajpendo.00620.2009 20215574 10.1152/ajpendo.00620.2009

[13] Cooksey RC, Jones D, Gabrielsen S, Huang J, Simcox JA, Luo B, et al Dietary iron restriction or iron chelation protects from diabetes and loss of beta-cell function in the obese (ob/ob lep-/-) mouse. American journal of physiology Endocrinology and metabolism 2010;298(6):E1236–43. doi: 10.1152/ajpendo.00022.2010 20354157 10.1152/ajpendo.00022.2010 PMC2886527

[14] Valenti L, Fracanzani AL, Dongiovanni P, Bugianesi E, Marchesini G, Manzini P, et al Iron depletion by phlebotomy improves insulin resistance in patients with nonalcoholic fatty liver disease and hyperferritinemia: evidence from a case-control study. The American journal of gastroenterology 2007;102(6):1251–8. doi: 10.1111/j.1572-0241.2007.01192.x 17391316 10.1111/j.1572-0241.2007.01192.x

[15] Festa M, Ricciardelli G, Mele G, Pietropaolo C, Ruffo A, Colonna A. Overexpression of H ferritin and up-regulation of iron regulatory protein genes during differentiation of 3T3-L1 pre-adipocytes. The Journal of biological chemistry 2000;275(47):36708–12. doi: 10.1074/jbc.M004988200 10978328 10.1074/jbc.M004988200

[16] Farahani P, Chiu S, Bowlus CL, Boffelli D, Lee E, Fisler JS, et al Obesity in BSB mice is correlated with expression of genes for iron homeostasis and leptin. Obesity research 2004;12(2):191–204. doi: 10.1038/oby.2004.26 14981211 10.1038/oby.2004.26

[17] Green A, Basile R, Rumberger JM. Transferrin and iron induce insulin resistance of glucose transport in adipocytes. Metabolism: clinical and experimental 2006;55(8):1042–5. 16839839 10.1016/j.metabol.2006.03.015

[18] Tanner LI, Lienhard GE. Insulin elicits a redistribution of transferrin receptors in 3T3-L1 adipocytes through an increase in the rate constant for receptor externalization. The Journal of biological chemistry 1987;262(19):8975–80. 3298247

[19] Ganz T. Macrophages and systemic iron homeostasis. Journal of innate immunity 2012;4(5–6):446–53. doi: 10.1159/000336423 22441209 10.1159/000336423 PMC6741611

[20] Morris DL, Singer K, Lumeng CN. Adipose tissue macrophages: phenotypic plasticity and diversity in lean and obese states. Current opinion in clinical nutrition and metabolic care 2011;14(4):341–6. doi: 10.1097/MCO.0b013e328347970b 21587064 10.1097/MCO.0b013e328347970b PMC4690541

[21] Lumeng CN, DelProposto JB, Westcott DJ, Saltiel AR. Phenotypic switching of adipose tissue macrophages with obesity is generated by spatiotemporal differences in macrophage subtypes. Diabetes 2008;57(12):3239–46. doi: 10.2337/db08-0872 18829989 10.2337/db08-0872 PMC2584129

[22] Corna G, Campana L, Pignatti E, Castiglioni A, Tagliafico E, Bosurgi L, et al Polarization dictates iron handling by inflammatory and alternatively activated macrophages. Haematologica 2010;95(11):1814–22. doi: 10.3324/haematol.2010.023879 20511666 10.3324/haematol.2010.023879 PMC2966902

[23] Orr JS, Kennedy A, Anderson-Baucum EK, Webb CD, Fordahl SC, Erikson KM, et al Obesity Alters Adipose Tissue Macrophage Iron Content and Tissue Iron Distribution. Diabetes 2014. 10.2337/db13-0213 PMC3900546 24130337

[24] Pasarica M, Sereda OR, Redman LM, Albarado DC, Hymel DT, Roan LE, et al Reduced adipose tissue oxygenation in human obesity: evidence for rarefaction, macrophage chemotaxis, and inflammation without an angiogenic response. Diabetes 2009;58(3):718–25. doi: 10.2337/db08-1098 19074987 10.2337/db08-1098 PMC2646071

[25] Ye J, Gao Z, Yin J, He Q. Hypoxia is a potential risk factor for chronic inflammation and adiponectin reduction in adipose tissue of ob/ob and dietary obese mice. American journal of physiology Endocrinology and metabolism 2007;293(4):E1118–28. doi: 10.1152/ajpendo.00435.2007 17666485 10.1152/ajpendo.00435.2007

[26] Mastrogiannaki M, Matak P, Keith B, Simon MC, Vaulont S, Peyssonnaux C. HIF-2alpha, but not HIF-1alpha, promotes iron absorption in mice. The Journal of clinical investigation 2009;119(5):1159–66. doi: 10.1172/JCI38499 19352007 10.1172/JCI38499 PMC2673882

[27] Shah YM, Matsubara T, Ito S, Yim SH, Gonzalez FJ. Intestinal hypoxia-inducible transcription factors are essential for iron absorption following iron deficiency. Cell metabolism 2009;9(2):152–64. doi: 10.1016/j.cmet.2008.12.012 19147412 10.1016/j.cmet.2008.12.012 PMC2659630

[28] Xue X, Ramakrishnan S, Anderson E, Taylor M, Zimmermann EM, Spence JR, et al Endothelial PAS domain protein 1 activates the inflammatory response in the intestinal epithelium to promote colitis in mice. Gastroenterology 2013;145(4):831–41. doi: 10.1053/j.gastro.2013.07.010 23860500 10.1053/j.gastro.2013.07.010 PMC3799890

[29] Sinke AP, Caputo C, Tsaih SW, Yuan R, Ren D, Deen PM, et al Genetic analysis of mouse strains with variable serum sodium concentrations identifies the Nalcn sodium channel as a novel player in osmoregulation. Physiol Genomics 2011;43(5):265–70. doi: 10.1152/physiolgenomics.00188.2010 21177381 10.1152/physiolgenomics.00188.2010 PMC3068516

[30] Rebouche CJ, Wilcox CL, Widness JA. Microanalysis of non-heme iron in animal tissues. Journal of biochemical and biophysical methods 2004;58(3):239–51. doi: 10.1016/j.jbbm.2003.11.003 15026210 10.1016/j.jbbm.2003.11.003

[31] Livak KJ, Schmittgen TD. Analysis of relative gene expression data using real-time quantitative PCR and the 2(-Delta Delta C(T)) Method. Methods 2001;25(4):402–8. doi: 10.1006/meth.2001.1262 11846609 10.1006/meth.2001.1262

[32] Ma X, Patterson KJ, Gieschen KM, Bodary PF. Are serum hepcidin levels chronically elevated in collegiate female distance runners? International journal of sport nutrition and exercise metabolism 2013;23(5):513–21. 23580449 10.1123/ijsnem.23.5.513

[33] Cinti S, Mitchell G, Barbatelli G, Murano I, Ceresi E, Faloia E, et al Adipocyte death defines macrophage localization and function in adipose tissue of obese mice and humans. Journal of lipid research 2005;46(11):2347–55. doi: 10.1194/jlr.M500294-JLR200 16150820 10.1194/jlr.M500294-JLR200

[34] Khan T, Muise ES, Iyengar P, Wang ZV, Chandalia M, Abate N, et al Metabolic dysregulation and adipose tissue fibrosis: role of collagen VI. Molecular and cellular biology 2009;29(6):1575–91. doi: 10.1128/MCB.01300-08 19114551 10.1128/MCB.01300-08 PMC2648231

[35] Dongiovanni P, Ruscica M, Rametta R, Recalcati S, Steffani L, Gatti S, et al Dietary iron overload induces visceral adipose tissue insulin resistance. The American journal of pathology 2013;182(6):2254–63. doi: 10.1016/j.ajpath.2013.02.019 23578384 10.1016/j.ajpath.2013.02.019

[36] Hazel M, Cooksey RC, Jones D, Parker G, Neidigh JL, Witherbee B, et al Activation of the hexosamine signaling pathway in adipose tissue results in decreased serum adiponectin and skeletal muscle insulin resistance. Endocrinology 2004;145(5):2118–28. doi: 10.1210/en.2003-0812 14684615 10.1210/en.2003-0812

[37] Gotardo EM, dos Santos AN, Miyashiro RA, Gambero S, Rocha T, Ribeiro ML, et al Mice that are fed a high-fat diet display increased hepcidin expression in adipose tissue. Journal of nutritional science and vitaminology 2013;59(5):454–61. 24418880 10.3177/jnsv.59.454

[38] Khor VK, Tong MH, Qian Y, Song WC. Gender-specific expression and mechanism of regulation of estrogen sulfotransferase in adipose tissues of the mouse. Endocrinology 2008;149(11):5440–8. doi: 10.1210/en.2008-0271 18669602 10.1210/en.2008-0271 PMC2584587

[39] Mattace Raso G, Irace C, Esposito E, Maffettone C, Iacono A, Di Pascale A, et al Ovariectomy and estrogen treatment modulate iron metabolism in rat adipose tissue. Biochemical pharmacology 2009;78(8):1001–7. doi: 10.1016/j.bcp.2009.05.034 19501056 10.1016/j.bcp.2009.05.034

[40] Khor VK, Dhir R, Yin X, Ahima RS, Song WC. Estrogen sulfotransferase regulates body fat and glucose homeostasis in female mice. American journal of physiology Endocrinology and metabolism 2010;299(4):E657–64. doi: 10.1152/ajpendo.00707.2009 20682840 10.1152/ajpendo.00707.2009 PMC2957869

