# Supplementary figures and images for: Iron elevation and adipose tissue remodeling in the epididymal depot of a mouse model of polygenic obesity
Source: PLoS One. 2017 Jun 26;12(6):e0179889. doi: 10.1371/journal.pone.0179889 (PMC5484604; doi:10.1371/journal.pone.0179889)

S1 Fig

A.

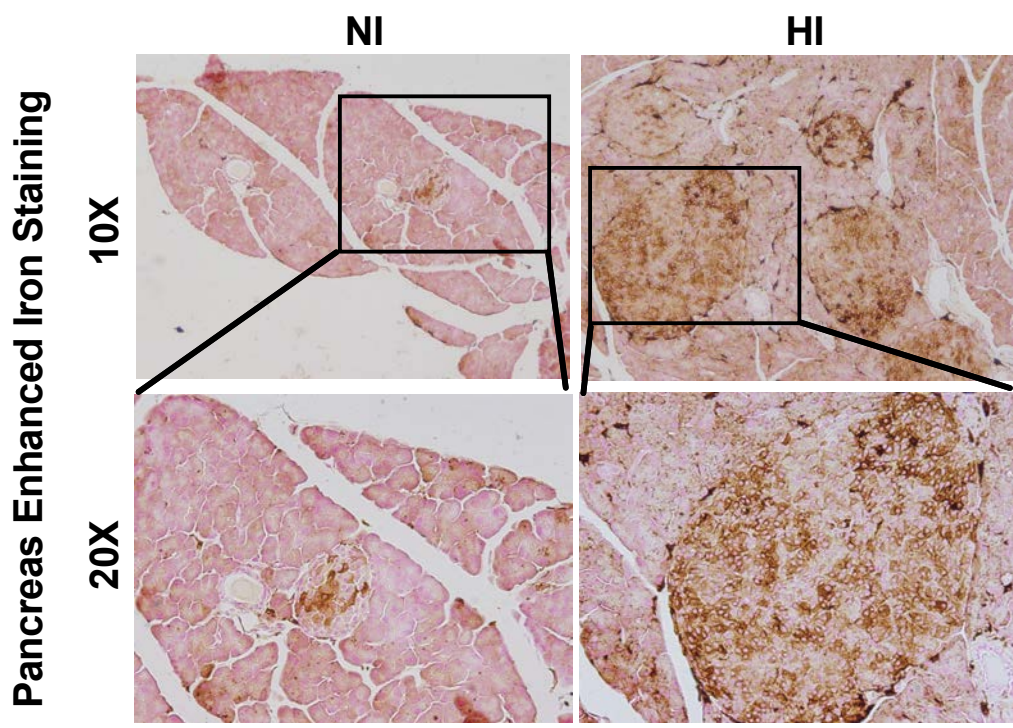

B.

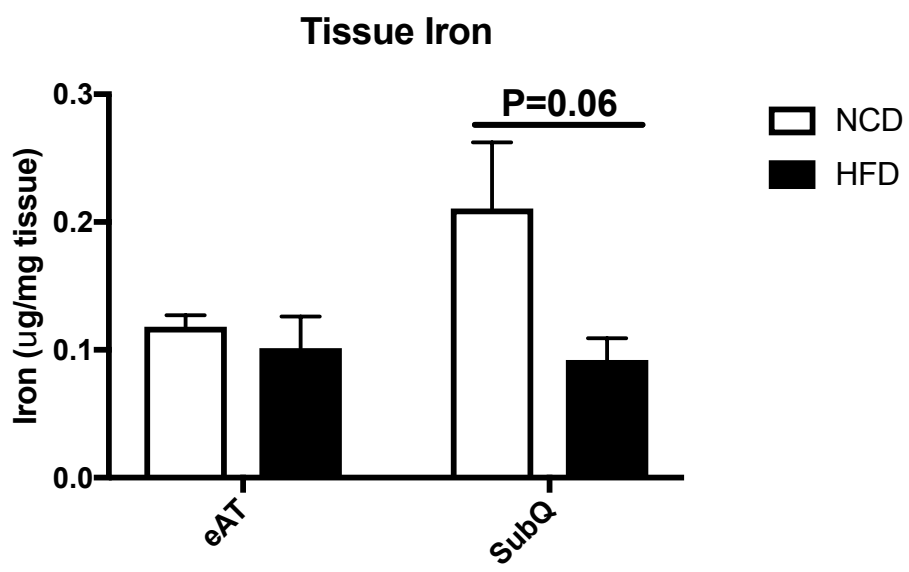

Supplement: S1 Fig — A) Perl’s Prussian Blue staining in the pancreas from HI and NI groups. Top panel represents pancreas from NI and HI groups with 10X magnification, and lower panel is with 20X magnification. B) Tissue iron levels in eAT and subcutaneous adipose tissue from 12-weeks NCD or HFD intervention C57BL/6J male mice. NCD (open bar) vs HFD (filled bar). Colors: iron, brown. (PDF) [file pone.0179889.s001.pdf]

S2 Fig

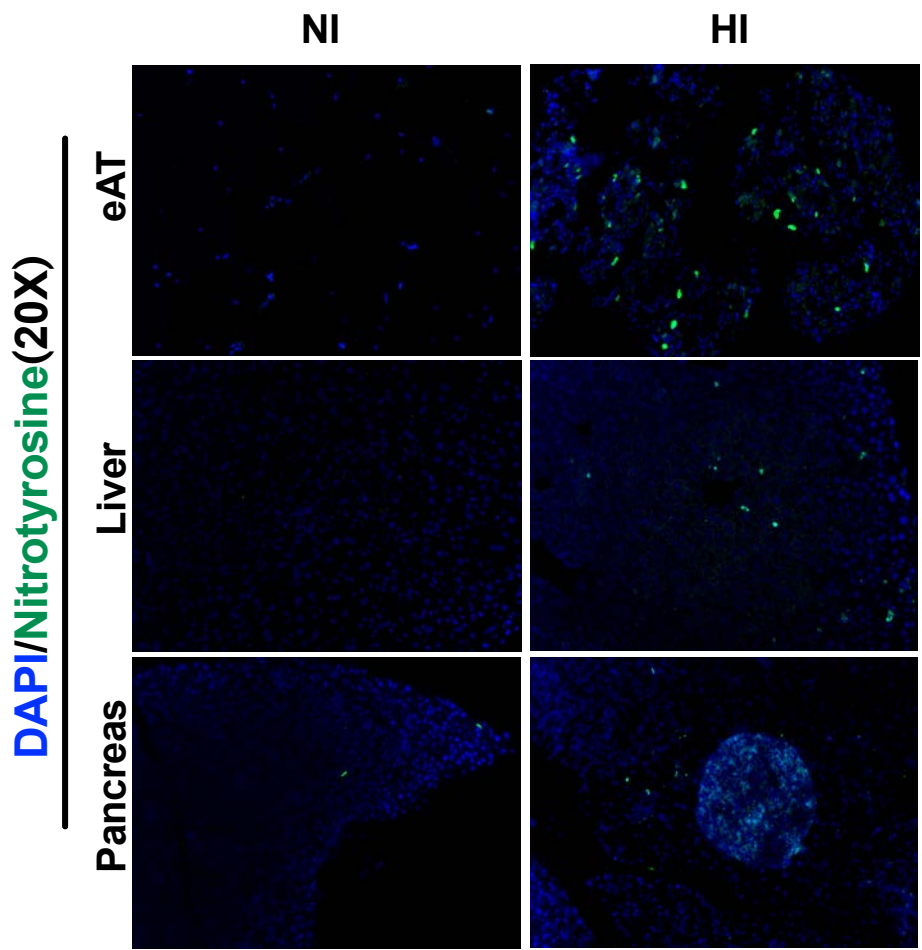

Supplement: S2 Fig — Immunoflorescent staining for Nitrotyrosine eAT, liver and pancreas in NI and HI groups. (PDF) [file pone.0179889.s002.pdf]
